# Clinical Outcomes After Arthroscopic Broström-Gould Procedure for Chronic Lateral Ankle Instability

**DOI:** 10.7759/cureus.81025

**Published:** 2025-03-23

**Authors:** Hasjmy Mohamad, Don Koh, Raj Socklingam, Darshana Chandrakumara, Ing How Moo, Charles Kon

**Affiliations:** 1 Orthopaedics, Changi General Hospital, Singapore, SGP

**Keywords:** anterior talofibular ligament, arthroscopic ankle surgery, broström-gould, chronic lateral ankle instability, lateral ankle sprain

## Abstract

Background

Chronic lateral ankle sprains are common injuries that are largely sequelae of inversion injuries of the ankle. These injuries are amenable to surgical intervention, namely, Broström-Gould reconstruction, which is commonly performed for the above injury. Many open and arthroscopic variations of the technique have been described. Arthroscopic Broström-Gould has been shown to have comparable outcomes to the open technique.

Analysis of 39 patients who underwent arthroscopic Broström-Gould at a tertiary institution showed excellent outcomes from all three functional scores.

Methods

Thirty-nine patients underwent arthroscopic Broström-Gould in 2021 at a tertiary institution and were followed up for up to one year postoperatively. All patients had failed conservative treatment and reported pain and chronic instability prior to surgical intervention. Outcomes were measured with functional scores, namely, the visual analogue scale (VAS), American Orthopaedic Foot and Ankle Society Ankle-Hindfoot Score (AOFAS), and Karlsson and Peterson Scoring System (K-P). Other demographics such as age, gender, laterality, and body mass index were also recorded.

Statistical analysis was performed with GraphPad Prism (GraphPad Software, San Diego, CA). Outcomes were analysed by paired t-test and statistical significance was set at p < 0.05.

Results

A total of 39 patients with chronic lateral ankle instability (CLAI) who underwent arthroscopic Broström-Gould were followed up for up to one year postoperatively. Demographics were as follows: 22 males and 17 females, 20 right ankles and 19 left ankles, average age of 36.2 years (19.6-64.8 years), and BMI of 27.4 (19.2-40).

At follow-up, functional scores were measured as described in the methods section above. Preoperative VAS scores were 5.87 ± 1.92 (1-10) while postoperative VAS scores were 2.0 ± 2.16 (0-7). Preoperative K-P scores were 43.68 ± 13.2 (7-70) while postoperative K-P scores were 69.21 ± 17.86 (37-100). Preoperative AOFAS scores were 62.53 ± 16.05 (29-83) while postoperative AOFAS scores were 83.8 ± 11.49 (59-100).

Conclusions

Significant improvement of all three functional scores (VAS, K-P, and AOFAS scores) following arthroscopic Broström-Gould was noted. This shows that the arthroscopic Broström-Gould is an effective method to treat CLAI with good outcomes.

## Introduction

Ankle sprains are the most common injury type of sprains and the most common type of injury in physically active individuals. Large registry-based studies in the US, UK, and Netherlands have reported incidence rates varying between 2.15 and 26.6 per 1000 person-years [1-4]. However, it is plausible that the actual incidence rates are likely underreported as many individuals do not present to emergency departments or large centres with these injuries.

Lateral ankle sprain consists of 85% of all ankle sprains [5] and is defined as an acute traumatic injury to the lateral ligament complex of the ankle joint as a result of excessive inversion of the rear foot or combines plantar flexion and adduction of the foot [6].

Chronic lateral ankle instability (CLAI) is characterised by a prior history of one significant ankle sprain for at least 12 months, with a history of the previously injured joint “giving way”, feelings of instability, and/or recurrent sprains. The proposed model by Hertel et al. describes acute primary tissue injury resulting in a series of pathomechanical, sensory-perceptual, and motor-behavioural impairments that result in the symptoms mentioned above [7]. A total of 20% of ankle sprains progress to CLAI [8]. These symptoms have an impact on physical function, quality of life, and economic burden on the population. Post-traumatic osteoarthritis of the ankle has also been proposed to be a sequela of CLAI [9].

The goal of surgical management for CLAI is to restore ankle stability in a previously injured ankle joint. The most widely used surgical technique is the Broström-Gould procedure, of which many adaptations to the procedure have been described over the years. Arthroscopic variations of the Broström-Gould have been gaining popularity and have been described in the literature. Variations in arthroscopic methods include a method of repair of the anterior talofibular ligament (ATFL) and surgical access [10,11].

This paper aims to present the outcomes of arthroscopic Broström-Gould at a tertiary institution.

## Materials and methods

All patients who underwent an Arthroscopic Broström-Gould procedure for CLAI between 2021 and 2022 at the authors' institution were identified via manual chart review. Patients who subsequently suffered injuries to the operated limb or were unable to be contacted for review were excluded from this paper.

Surgical procedure

The patient was positioned supine under general or regional anaesthesia, followed by meticulous cleaning. A thigh tourniquet was inflated prior to the initiation of the surgery. Saline insufflation of the ankle joint and standard anteromedial portal anterolateral portals were created.

A diagnostic scope with a Smith and Nephew 4.0 mm arthroscope (Smith & Nephew, London, UK) was performed to assess the medial compartment of the ankle joint. Treatment of any medial compartment impingement was carried out by adequate arthroscopic debridement.

Subsequently, the lateral compartment was assessed. Adequate debridement of the ATFL and surrounding synovitis was performed. Following that, the arthroscopic Broström-Gould procedure was performed by anchoring the AFTL to the distal fibula with SutureFIX suture anchors (Smith & Nephew, London, UK). Typically two anchors are used depending on the quality of the ATFL stump. The resulting stabilisation is then tested intraoperatively by an anterior drawer test.

Patients are managed postoperatively with immediate weight bearing and a full range of motion with an ankle brace. The ankle brace is removed at six weeks postoperatively. They are reviewed by physiotherapists on postoperative day one and followed up as an outpatient. During outpatient follow-up that was carried out at two weeks, six weeks, three months, and up to one year, the patient's wound and ankle stability were assessed to assess for wound complications or recurrent instability, along with functional assessment scores.

Functional scores

Clinical outcomes were objectively measured using the various functional scores discussed below. Results were considered significant if p-values were less than 0.05.

Visual Analogue Scale (VAS) Score

VAS score is calculated based on a self-reported measure of pain, expressed on a continuum on a scale of 0-10 where 0 represents no pain and a score of 10 represents the maximum amount of pain.

Karlsson and Peterson Scoring System

It uses a subjective scoring scale of eight components (pain, swelling, instability, stiffness, stair climbing, running, work activities, and support) to assess overall ankle function and stability. It is scored from 0 to 100, where a higher score indicates better function.

American Orthopaedic Foot and Ankle Society Ankle-Hindfoot Score (AOFAS)

It combines the use of subjective and objective scores (pain, function, alignment) to a maximum score of 100 to assess and quantify clinical ratings, where a higher score indicates better function.

Data management and statistical analysis

Information collected during the course of our research was meticulously recorded and stored in offline servers at our institution as encrypted files to ensure data security.

An individual statistician conducted statistical analysis, using the GraphPad Prism (v.6.04) software (GraphPad Software, San Diego, CA). P-values were calculated and extracted using the paired sample t-test. The mean, standard deviation, and confidence intervals were calculated, and graphs were plotted to represent the data.

## Results

Demographics

In 2021, 39 patients had arthroscopic Broström-Gould reconstruction in our institution. Of these, 22 patients (56%) were male and 17 patients (44%) were female. Twenty patients (51%) had an operation on the right ankle and 19 patients (49%) had an operation on the left ankle. The mean age of this cohort was 36.2 years with a range of 19.6 to 64.8 years. The mean BMI was 27 kg/m2 with a range of 19.2 to 40 kg/m2. This is illustrated in Table 1. Of this cohort, no reports of complications or recurrent instability that required revision surgery were noted.

**Table 1:** Demographics of 39 patients in the cohort. Gender and laterality values are given as N (%). Age and BMI values are given as mean ± standard deviation (range).

Demographic		Value
Gender	Male	22 (56)
	Female	17 (44)
Laterality	Right	20 (51)
	Left	19 (49)
Age (years)		36.2 ± 14.46 (19.6-64.8)
BMI (kg/m^2^)		27.4 ± 6.04 (19.2-40)

Functional scores

Our results show the functional scores (VAS, Karlsson-Peterson, AOFAS) significantly improved postoperatively, as illustrated in Table 2. The mean preoperative VAS score was 5.87, with a standard deviation of 1.92 and a range of 1-10, while the mean postoperative VAS score was 2.0, with a standard deviation of 2.16 and a range of 0-7. The mean preoperative Karlsson-Peterson score was 43.68, with a standard deviation of 13.2 and a range of 7-70, while the mean postoperative Karlsson-Peterson score was 69.21, with a standard deviation of 17.86 and a range of 37-100. The mean preoperative AOFAS score was 62.53, with a standard deviation of 16.05 and a range of 29-83, while the mean postoperative AOFAS score was 83.8, with a standard deviation of 11.49 and a range of 59-100.

**Table 2:** Functional scores of a cohort of 39 patients. The values are given as mean ± standard deviation (range). VAS: visual analogue scale; AOFAS: American Orthopaedic Foot and Ankle Society Ankle-Hindfoot Score.

Functional score	Preoperative	Postoperative	t score	p-value
VAS	5.87 ± 1.92 (1-10)	2.0 ± 2.16 (0-7)	8.75	<0.0001
Karlsson-Peterson score	43.68 ± 13.2 (7-70)	69.21 ± 17.86 (37-100)	-8.71	<0.0001
AOFAS	62.53 ± 16.05 (29-83)	83.8 ± 11.49 (59-100)	-5.91	<0.0001

## Discussion

Methods of addressing lateral ankle instability

A plethora of methods for addressing lateral ankle instability is present. Variations of open techniques and arthroscopic techniques have been described in the literature; however, the Broström-Gould has been considered the gold standard procedure.

Arthroscopic procedures have been increasing in popularity, replacing open Broström-Gould [12] or open reconstruction procedures [13].

Arthroscopic procedures generally have steeper learning curves than open procedures. A recent study by Zhao et al. described an increased learning curve cutoff for arthroscopic Broström-Gould at 22 cases over 12 cases for open Broström-Gould with no significant differences between clinical function scores assessed in the learning and proficiency groups [14]. Clinical outcomes in both groups were similar apart from arthroscopic procedures having superior VAS scores, postoperative weight-bearing times, and hospitalisation.

Advantages of arthroscopic ankle procedures

Arthroscopic Broström-Gould has its own advantages over open surgery and has been shown to have superior pain and functional outcomes [15].

One advantage of arthroscopic evaluation over open surgery of the ankle is that it allows thorough evaluation of the ankle joint, where concomitant pathologies may be present. A retrospective study by Koh et al. evaluated the incidence of ligamentous, tendon, and other structural injuries that were associated with ATFL injuries based on MRI findings [16]. The pathologies reported are the calcaneofibular ligament, deltoid ligament, peroneal tendon, and osteochondral defects.

Furthermore, an arthroscopic approach will allow treatment of some of the above concomitant pathologies in the same setting such as impingement and osteochondral lesions [17].

Arthroscopic repair has also been shown to have better early outcomes than open repair; however, long-term outcomes were similar. Matsui et al. described improved VAS scores at three days for arthroscopic repair over the open repair and a shorter time to return to daily life [18]. This would likely affect not only patient satisfaction but also encourage earlier compliance to post-rehabilitation therapy.

Disadvantages of arthroscopic ankle procedures

Complications relating to arthroscopic ankle surgery have been described in the literature. The most commonly described complications in the literature are neurovascular injuries and wound complications, among others such as type 1 complex regional pain syndrome, pseudoaneurysms, compartment syndrome, and peroneal tendon irritation [19].

Localized superficial infections have been described as complications following ankle arthroscopy [19]. However, in a meta-analysis by Moorthy et al., significantly lower rates of wound-related complications were noted in arthroscopic Broström-Gould procedures compared to open Broström-Gould procedures [20].

Nerve injuries have been reported following ankle arthroscopy, such as superficial peroneal nerve (SPN), sural nerve, saphenous nerve, and deep peroneal nerve. In particular, injuries to the SPN are the most commonly reported nerve injury [21]. This is primarily due to the position of the anterolateral portal, which is commonly placed just lateral to the peroneus tertius tendon.

Over the years of arthroscopic ankle surgeries, multiple variations of techniques and variations in steps have been described to reduce the rate of the above complications. Several developments in anterior ankle arthroscopy have been described, such as non-invasive distraction techniques, dorsiflexion method [22], identification of subcutaneous vasculature [23], and identification of closely related structures such as the immediate dorsal cutaneous nerve and medial dorsal cutaneous nerve. Danger zones have been described in the literature illustrating areas to avoid during ankle arthroscopic procedures to prevent injury to the SPN [24].

Rehabilitation and return to sport

Wide variations in postoperative rehabilitation have been described in the literature, with varying progressive ranges of motion and weight bearing [25]. In our paper, we employ immediate weight bearing with an ankle brace as part of postoperative management, with eventual removal of the brace at six weeks. Recent studies have shown that early weight bearing has been shown to improve the rate of recovery post surgical intervention [26,27].

CLAI has a high incidence rate in highly active populations [28]. Hence, return to sport is an important outcome for these patients. Studies have shown a significant proportion of patients returning to sport postoperatively to the same level or higher [29,30]. Positive factors from these studies include greater preoperative motivation and participation in elite sports whereas negative factors include high BMI and increasing age.

Limitations

Limitations of this study include a small sample size in a singular institution performed by a single operator. As such, variability between the demographics would be difficult to assess, for example, variations in BMI and co-morbidities that would positively or negatively affect outcomes. Furthermore, measured outcomes are subjective as they are largely patient reported and would be subject to bias.

Moving forward, long-term follow-up and outcomes can be assessed such as return to sport or intense physical activities, burden on healthcare systems, and quality of life. Long-term follow-up is required with the evaluation of the incidence of known potential sequelae such as the development of ankle osteoarthritis. Further evaluation of concomitant pathologies that can be addressed in the same setting would also be beneficial to evaluate the necessity of intervention.

## Conclusions

Significant improvement of all three functional scores (VAS scores, Karlsson-Peterson scores, and AOFAS scores) following arthroscopic Broström-Gould was noted. This shows that the arthroscopic Broström-Gould is an effective method to treat CLAI with good outcomes.
